# Point-of-Care Diagnostic Devices for Detection of *Escherichia coli* O157:H7 Using Microfluidic Systems: A Focused Review

**DOI:** 10.3390/bios13070741

**Published:** 2023-07-17

**Authors:** Naseem Abbas, Sehyeon Song, Mi-Sook Chang, Myung-Suk Chun

**Affiliations:** 1 Department of Mechanical Engineering, Sejong University, Gwangjin-gu, Seoul 05006, Republic of Korea; naseem.abbas@sejong.ac.kr; 2 Laboratory of Stem Cell & Neurobiology, Department of Oral Anatomy & Dental Research Institute, Seoul National University School of Dentistry, Jongno-gu, Seoul 03080, Republic of Korea; songneuron@snu.ac.kr; 3 Interdisciplinary Program in Neuroscience, Seoul National University College of Natural Sciences, Gwanak-gu, Seoul 08826, Republic of Korea; 4 Sensor System Research Center, Advanced Materials Research Division, Korea Institute of Science and Technology (KIST), Seongbuk-gu, Seoul 02792, Republic of Korea; 5 Biomedical Engineering Division, KIST School, University of Science and Technology, Seoul 02792, Republic of Korea

**Keywords:** *E. coli* O157:H7, microfluidics, point-of-care, antimicrobial peptide, pathogen, biosensing

## Abstract

Bacterial infections represent a serious and global threat in modern medicine; thus, it is very important to rapidly detect pathogenic bacteria, such as *Escherichia coli* (*E. coli*) O157:H7. Once treatments are delayed after the commencement of symptoms, the patient’s health quickly deteriorates. Hence, real-time detection and monitoring of infectious agents are highly critical in early diagnosis for correct treatment and safeguarding public health. To detect these pathogenic bacteria, many approaches have been applied by the biosensors community, for example, widely-used polymerase chain reaction (PCR), enzyme-linked immunosorbent assay (ELISA), culture-based method, and adenosine triphosphate (ATP) bioluminescence. However, these approaches have drawbacks, such as time-consumption, expensive equipment, and being labor-intensive, making it critical to develop ultra-sensitive and highly selective detection. The microfluidic platform based on surface plasmon resonance (SPR), electrochemical sensing, and rolling circle amplification (RCA) offers proper alternatives capable of supplementing the technological gap for pathogen detection. Note that the microfluidic biochip allows to develop rapid, sensitive, portable, and point-of-care (POC) diagnostic tools. This review focuses on recent studies regarding accurate and rapid detection of *E. coli* O157:H7, with an emphasis on POC methods and devices that complement microfluidic systems. We also examine the efficient whole-body detection by employing antimicrobial peptides (AMPs), which has attracted growing attention in many applications.

## 1. Introduction

Ingesting contaminated food such as chicken, beef, milk, juice, vegetables, and tap water not only endangers human health but also results in significant financial losses, posing a serious threat to public health [1]. Among the pathogenic bacteria, *Escherichia coli* (*E. coli*) O157:H7 is a quite important foodborne or waterborne infectious pathogen of global concern, as well as the most common and lethal serotype of enterohemorrhagic *E. coli* [2]. In addition, contamination of water sources with this pathogenic bacteria is a big problem in both developing and developed countries [3]. Infants, children, immunocompromised individuals, and the elderly are the most vulnerable to waterborne infections. Ingestion of as few as 10 organisms can result in life-threatening symptoms, especially in young and immunocompromised people [4,5,6]. Majowicz et al. [7] published a systematic review of outbreak surveillance data on the global prevalence of Shiga-toxin generating *E. coli* infections and calculated that over 2.8 million people are affected each year. Between 1998 and 2017, there were approximately 350 occurrences of *E. coli* O157:H7 in more than 45 US states, Canada, the United Kingdom, Japan, and Ireland. More than half of all outbreaks (54%) were caused by contaminated water and food. In addition to the safety issues related to food, it has also become a significant medical and global public health problem. Therefore, establishing the effective and rapid detection of *E. coli* O157:H7 is really critical for diagnostic platform. Developing a microfluidic device that is ultra-sensitive, fast, highly selective (specific), easy to use, and portable as valuable proof-of-concept should be a challenge for public health [8].

Fortunately, several advanced technologies have been developed for the efficient detection of *E. coli* O157:H7. So far, its clinical detection has relied on standard approaches, such as real-time PCR [9,10,11], antibody-based detection, and the well-known ELISA method [12,13]. Antibody-based detection (immunoassay) is limited by low sensitivity and dependence on specific antibodies. PCR, which amplifies specific DNA sequences unique to *E. coli* O157:H7, is a commonly used method. This technique enables rapid and sensitive detection of bacteria in food samples or clinical specimens. PCR-based assays can provide results within hours, making them invaluable for timely identification and response to potential outbreaks. PCR has been widely used in laboratory settings for several decades, significantly contributing to advancements in various fields, including molecular biology, medical diagnostics, and forensic sciences. Its established methodology and robustness have made it the gold standard for many applications. However, when comparing conventional PCR to point-of-care (POC) devices, multiple parameters were considered including time and speed, portability, accessibility, sensitivity and specificity, and cost and affordability. Conventional PCR has a proven track record of high sensitivity and specificity, allowing for accurate detection and quantification of nucleic acids.

While POC devices may not always match the sensitivity of conventional PCR, recent advancements have significantly improved their performance. It is worth noting that point-of-care devices are often designed to target specific pathogens or genetic markers, optimizing their sensitivity and specificity for the intended diagnostic purpose. Similarly, POC devices are typically portable, compact, and easy to use, making them suitable for deployment in resource-limited settings, remote areas, or during emergencies. In contrast, conventional PCR machines are larger, stationary instruments that require specialized laboratory infrastructure and trained personnel. The accessibility and convenience of POC devices broaden their potential applications and impact.

Another innovative technology for *E. coli* O157:H7 detection is immunological assays, such as ELISA and lateral flow immunoassays (LFIA). These tests utilize antibodies that specifically bind to *E. coli* O157:H7 antigens, allowing for the detection of the bacteria in various samples. Immunoassays are relatively quick, cost-effective, and can be performed in the field, making them suitable for rapid screening purposes. In addition, biosensors have emerged as promising tools for the detection of *E. coli* O157:H7. Biosensors employ biological components, such as antibodies or DNA probes, coupled with transducers to convert the presence of bacteria into measurable signals. These devices offer real-time monitoring, high sensitivity, and portability, facilitating on-site testing and enabling early identification of contaminated sources. Lastly, advancements in genomic sequencing technologies have revolutionized the detection of *E. coli* O157:H7. Whole-genome sequencing (WGS) allows for comprehensive analysis of the bacteria’s genetic material, enabling the identification of specific virulence factors and tracing outbreaks back to their sources. WGS provides valuable insights into the epidemiology and evolution of *E. coli* O157:H7 strains, aiding in the development of targeted prevention and control strategies. WGS offers in-depth genetic analysis and is primarily used for long-term surveillance and research purposes. On the other hand, POC detection provides rapid, on-site testing, enabling quick decision-making and response to *E. coli* O157:H7 contamination events. Furthermore, it is necessary to design and fabricate a highly precise and cost-effective pathogenic bacteria diagnostic test that can be easily utilized in underdeveloped nations to combat infectious bacterial/viral infections without causing financial burden. POC devices aimed at clinical diagnostics are a good example of an interface where biomedical and engineering disciplines meet [14,15,16].

The incorporation of leading-edge technology into the microfluidic platform may provide very useful solutions for the relevant issues of diagnostic devices. A microfluid-based system offers advantages of relatively simple operation, low cost, multiple-target testing, ease of automation, portability, and compactness. As a consequence, surface plasmon resonance (SPR), electrochemical, and rolling circle amplification (RCA) have been revitalized due to rapid analysis, highly sensitive and selective performance, and consistent detection limit. Furthermore, these methods incorporated with a microfluidic system can be used in any location or POC, even if there is no electricity accessible, due to the ubiquitous concept.

This review focuses on the detection of *E. coli* O157:H7 using microfluidic systems, such as POC diagnostic devices including SPR, electrochemical sensing, and RCA. The most important performances for these methods are the limit of detection (LOD) and the detection time. The LOD and detection time were compared with each other from recent literature information, and then the best results of LOD and detection time were discussed for the detection of pathogenic bacteria. In addition, this review also presents the actually advanced *E. coli* detection by employing antimicrobial peptides (AMPs), which were introduced as emerging alternatives to antibodies. Among the different types of POC, AMP-based microfluidic platforms for pathogen sensing have gained much attention in view of whole-body detection with selectively binding affinity.

## 2. SPR-Based *E. coli* O157:H7 Detection

**Figure 1:**
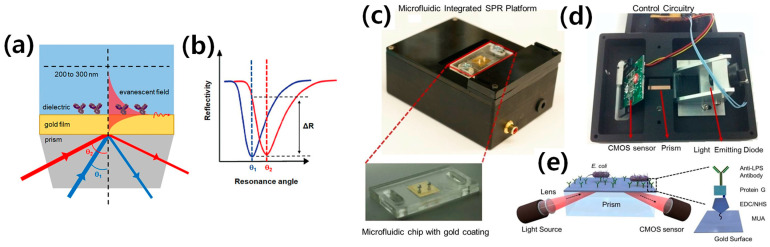
The principle of SPR as a label-free biosensing technology and SPR-based microfluidic platform for *E. coli* detection as a POC. (**a**) The incident light travels along the dielectric interface and excites plasmon at this interface. (**b**) Accurate binding of a biomolecule (antibody) on the surface of a gold film results in a shift in SPR resonance angle with before antibody binding θ_1_ (blue) and after binding θ_2_ (red). The occurrence of binding is denoted as the reflectivity angle (ΔR) with the measured resonance angle fixed. Reprinted with permission from [26]. (**c**) The disposable microfluidic chips, which are surface activated, are placed on the top of the device. (**d**) The electronic configuration of the device is depicted from bottom to top. A light-emitting diode (LED) illuminates a cylindrical lens, which focuses the light onto a rectangular prism. The reflected light is captured by a CMOS sensor, and the image is transmitted to a portable computer via control circuitry. (**e**) The schematic shows a microfluidic integrated SPR platform. Reprinted with permission from [24].

SPR-based biosensors are rapidly emerging as a direct label-free technology for the rapid and highly sensitive detection of chemical and biological analytes in critical fields, such as medical diagnostics, food safety, and environmental sensing, due to the unique optical properties they possess [17,18,19]. SPR utilizes the simple phenomenon of refractive index changes in conjunction with the binding of target biomolecules (either antigens or protein samples) to identify these changes. The SPR phenomena occur due to the excitement and collective vibration of free electrons in metal and dielectric junctions. This excitation is caused by the interaction of electromagnetic waves in the visible area with the plasmon of gold and silver nanoparticles [20,21,22]. When combined with well-developed microfluidic technology, SPR offers numerous advantages, which are explained and summarized in the concluding section of this review article. The complete binding mechanism is illustrated in Figure 1a,b, where the binding event is indicated by a change in reflectivity ΔR at a fixed observed angle. Before antibody binding, θ_1_ (blue) is shown in both panels (panel A and panel B), while after the binding of antibody, θ_2_ (red) is observed. The change in reflectance ΔR represents the binding event or sensitivity at the fixed observed angle. In the 1990s, the United States Department of Agriculture (USDA) introduced a commercial SPR apparatus for the detection of *E. coli* O157:H7, where the antibodies were sufficiently immobilized on the surface of a substrate. Since then, SPR has become a popular label-free tool in the biosensors society for detecting a variety of chemical and biological samples [23], serving as a POC technique in microfluidics.

Numerous researchers have worked in the SPR domain as a POC diagnostic device for the accurate, rapid, and highly sensitive detection of *E. coli* O157:H7 with no cross-reaction. To achieve this goal, Tokel et al. [24] developed a microfluidic SPR biochip capable of detecting and quantifying *E. coli* and *Staphylococcus aureus*. A microfluidic channel with a volume of 4 µL was created by laser cutting a microchannel form in a double-sided adhesive (DSA) layer with a thickness of 50 μm. The DSA layer held together the polymethyl methacrylate (PMMA) layer and the gold-coated glass substrate. The sample was injected into the channel and adhered to the gold surface after opening a single inlet and outflow port on the PMMA layer. For SPR measurement, the prism was placed on the PMMA-DSA-glass setup. Figure 1c,d depicts the custom-made SPR platform for microfluidic integration based on a complementary metal–oxide–semiconductor (CMOS) sensor. The lower part of Figure 1c shows an enlarged view of the microfluidic chip with a gold coating. Figure 1e illustrates the schematic of the microfluidic integrated SPR system, in which the gold surfaces were modified with different activators, including 11-mercaptoundeconoic acid (MUA), N-(3-dimethylaminopropyl)-N′-ethylcarbodiimide hydrochloride (EDC), N-hyroxysuccinimide (NHS), and anti-liposaccharide (LPS) antibodies, to capture *E. coli*. The antibodies in the microchannel capture bacteria, resulting in a change in the local refractive index. This change is detected by the reflected light, collected by the sensor, and delivered to a computer for analysis. The microfluidic SPR sensor was used to investigate the capture of *E. coli* with a LOD of 10^6^ CFU/mL. A detection time of 20 min was observed using this POC microfluidic system, which is considered a good reaction/detection time in biosensor design and fabrication. Previous studies have reported a lower LOD of 94 CFU/mL with SPR, which is approximately 10^4^ times better than the previous system [24,25]. Table 1 summarizes the most recent studies, including various parameters such as material type, method, LOD, detection time, detection range for linear analysis (to calculate R^2^ value using regression analysis), sample type, and sensor response.

In recent years, numerous studies have aimed at developing SPR biosensors for the fast and accurate detection of *E. coli* O157:H7. However, these studies often demonstrate the ability to identify bacterial pathogens only at higher concentrations and longer incubation times using expensive equipment, which is inadequate for most hazardous infections.

According to the literature, several assays have been employed for the detection of *E. coli* using the SPR method, including monoclonal antibody, polyclonal antibody, T4 bacteriophage, and AMP with magainin 1-C-based SPR detection. Each method possesses its own advantages and disadvantages, depending on factors such as the LOD, detection time, cost, and commercialization feasibility. The minimum detection time achieved was 10 min using a polyclonal antibody (rabbit anti-goat IgG), while a detection time of 120 min was observed when utilizing a chicken antibody for *E. coli* detection. Furthermore, the best LOD achieved using the SPR method was 10 CFU/mL. This notable achievement was made possible by detecting *E. coli* using murine anti-*E. coli* O157:H7 monoclonal antibodies, utilizing magnetic nanoparticles coated with an Au shell. In conclusion, SPR-based detection of *E. coli* O157:H7 offers a sensitive and real-time approach for the identification and quantification of the bacteria. This advancement contributes to enhanced food safety measures and improved public health.

The specificity of *E. coli* detection plays a crucial role in SPR-based biosensors. When specific antibodies or aptamers designed to recognize and bind to *E. coli* antigens are employed as capture molecules in an SPR assay, the specificity can be significantly high. These capture molecules are carefully selected to have minimal cross-reactivity with other bacterial species or contaminants, ensuring their specific interaction with the target *E. coli* bacteria. Numerous researchers have investigated the specificity of *E. coli* O157:H7 using the SPR method. For instance, Li et al. [27] determined the specificity of *E. coli* O157:H7 against *E. coli*, *S. typhimurium*, *S. aureus*, and *V. parahemolyticus*. They evaluated the signal intensities and found that the intensities associated with *E. coli* O157:H7 were almost five-fold stronger than those of the non-target pathogens. The most recent studies that focused on accurate detection using the SPR method are summarized in Table 2.

## 3. Electrochemical-Based *E. coli* O157:H7 Detection

**Figure 2:**
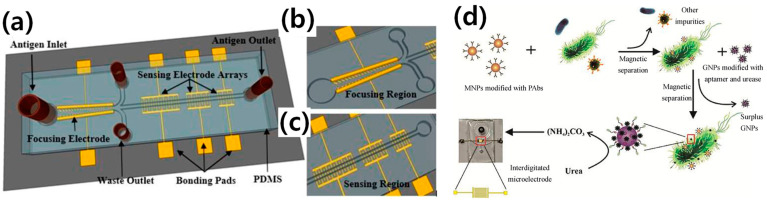
The schematic of (**a**) the impedance-based microfluidic biosensor for accurate and rapid detection of *E. coli* O157:H7, where the top cover is made of PDMS flexible material. The device features one inlet port through which the antigen passes and one outlet port through which the antigen exits after the reaction. (**b**) The focusing region is magnified to enhance clarity. (**c**) The microfluidic arrays consist of a series of sensing regions with gold electrodes embedded under epoxy-based negative photoresist (SU8) microchannels. Reprinted with permission from [37]. (**d**) The principle of the microfluidic impedance biosensor enables continuous-flow pathogen detection. Reprinted with permission from [38].

Electrochemical biosensors are commonly produced and widely employed for the detection of food-borne and water-borne pathogens due to the possibilities of miniaturization and the ability to build disposable, flexible, and affordable sensing systems. Over the last decade, various methodologies have been developed for the rapid quantification of *E. coli* O157:H7. According to the International Union of Pure and Applied Chemistry (IUPAC), an electrochemical biosensor is a self-contained device that utilizes a bioreceptor in conjunction with an electrochemical transduction component to provide quantitative or semi-quantitative analytical data [36]. Many researchers have made efforts to detect *E. coli* O157:H7 as a POC device using electrochemical methods. For this purpose, Dastider et al. [37] developed a microfluidic platform for accurate and sensitive detection, known as the MEMS sensor, which operates on the principle of dielectrophoresis force. The key components of this MEMS device are the design of the focusing region and the sensing region. The focusing region generates a positive dielectrophoresis force that moves the cells to the edges of the tilted thin-film electrode fingers in the center of the microchannel. The fluidic drag force then transports the focused cells to the sensing region, which contains multiple pairs of three interdigitated electrode arrays (IDEAs) embedded inside the microchannel. This technique allows for highly concentrated samples in the sensing region. For specific sensing of *E. coli* 0157:H7, the sensing IDEAs are functionalized with an anti-*E. coli* antibody. When *E. coli* binds to the antibody, there is an impedance change. The complete design of this MEMS microfluidic biosensor is shown in Figure 2. The detection time is 120 min and the LOD is 39 CFU/mL. Despite the good LOD, the long detection time makes this device less efficient for a POC application.

In addition, to further improve the LOD and detection time for accurate detection, Yao et al. [38] designed and fabricated a microfluidic biosensor based on electrochemical impedance spectroscopy (EIS) combined with magnetic nanoparticles (MNPs). MNP–bacteria–GNP–urease complexes were created by combining MNP–bacteria with gold nanoparticles (GNPs) modified with urease and aptamers against *E. coli* O157:H7. The complete development is shown in Figure 2d. A satisfactory linear relationship between the relative rate of impedance change of the catalysate and the concentration of the bacteria was established with a low detection limit of 12 CFU/mL and a detection period of 15 min. This represents an excellent LOD and detection time for POC application. The most recent studies on the detection of *E. coli* O157:H7 are summarized in Table 3, including the LOD, detection time, material type, methodology, assay structure, sample type, and detection range for linearity checking (R^2^ using regression analysis). In addition, the most recent studies that have focused on the accurate detection (specificity) using electrochemical methods are summarized in Table 4.

## 4. RCA-Based *E. coli* O157:H7 Detection

Rolling circle amplification (RCA) driven by DNA polymerase is an effective enzymatic isothermal process. It involves the synthesis of long single-stranded (ss) DNA molecules on a short circular ssDNA template using a single DNA or RNA primer. In addition, RCA can replicate circularized oligonucleotide probes with either linear or geometric kinetics. By modifying the template, introducing functional sequences during the reaction, and hybridizing the RCA products to oligonucleotides with specific functions, RCA can serve multiple purposes. Due to these innovative features, RCA-based nanotechnology has found wide applications in biological detection, medication delivery, and other fields [52]. A brief description of the RCA is as follows: First, a primer is conjugated to the necessary surface by employing surface activation and functionalization. The primer is then hybridized with a padlock probe, which is carefully designed to include a primer-binding site, a pathogen-binding site, and a self-assembly area that generates a molecular dumbbell shape. By regulating the length of each binding area, the padlock probe is designed to possess thermal stability at room temperature and specificity for primer and pathogen hybridization. After annealing, the padlock probe takes an asymmetric dumbbell shape. Subsequently, the probe is hybridized with the primer immobilized on the required surface. Hybridization occurs when the padlock probe comes into contact with a target pathogen. The opening padlock probe is ligated with a ligase to create a closed-loop template that can be utilized in the RCA procedure. During the RCA process, complementary single-stranded DNA gradually extends in a dumbbell form over time by DNA polymerase. The amplification of long DNAs, owing to the dumbbell-shaped template, leads to their easy entanglement with each other, resulting in aggregation with surrounding DNAs and the formation of a DNA gel. This DNA hydrogel has been developed for the visualized, simple, and rapid detection of *E. coli* O157:H7.

**Figure 3:**
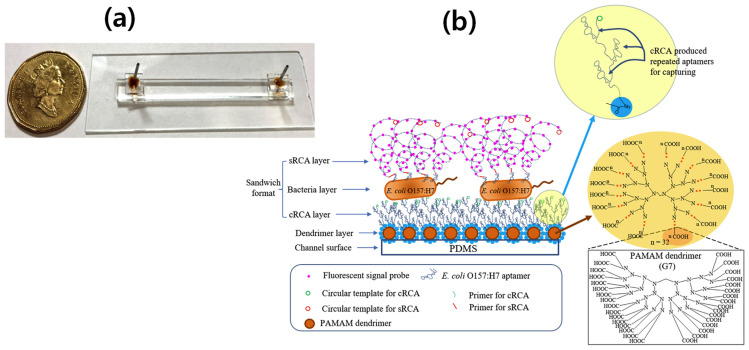
The principle of a microfluidic biosensor based on dual RCA for the whole cell detection of *E. coli* O157:H7. (**a**) An actual image of the microfluidic detection platform shows a single inlet and outlet ports. (**b**) A schematic illustrates a sandwich detection system where the surface of a microfluidic channel is modified with polyamidoamine (PAMAM) dendrimers. In situ capturing RCA (cRCA) is performed to generate repeating aptamers that capture the target cells. Subsequently, detection signals are amplified using signaling RCA (sRCA). The immobilized dendrimers on the PDMS surface provide several handles, allowing for more copies and higher density of dispersed cRCA on the capturing surface. Reprinted with permission from [53].

To achieve this goal, many researchers have made efforts to design and develop microfluidics RCA sensors for the rapid and accurate detection of *E. coli* O157:H7. Recently, Jiang et al. [53] developed an aptamer-based microfluidic platform for the detection of whole cells by applying the dual RCA technique. The complete design of dual-RCA microfluidic biosensor design is illustrated in Figure 3. The detection time and LOD are 120 min and 39 CFU/mL, respectively, which are better results than those with an incubation time of 120 min and LOD of 4.0 × 10^2^ CFU/mL [54]. The most recent studies on *E. coli* detection, including LOD and detection time, material type, methodology, assay structure, and sample type, are summarized in Table 5. Further exploration is still needed for the identification/detection of *E. coli* in order to improve the detection time and LOD.

PCR, the gold standard method, amplifies specific DNA sequences unique to *E. coli* O157:H7, enabling rapid and sensitive detection of the bacteria in food samples or clinical specimens. PCR-based assays provide results within hours, making them invaluable for timely identification and response to potential outbreaks. PCR has been extensively employed in laboratory settings for several decades, leading to significant advancements across various fields. When comparing RCA and PCR, both techniques serve as powerful molecular methods for nucleic acid amplification. While PCR is widely established and extensively used, RCA offers distinct advantages in specific applications. The following section outlines a comparative analysis of RCA and PCR, highlighting the advantages of RCA:

▪ Amplification Mechanism: The fundamental amplification mechanisms of RCA and PCR differ. PCR utilizes a thermal cycling process to amplify a specific DNA segment, employing heat-stable DNA polymerases. On the other hand, RCA employs a rolling circle mechanism, where a circular template is exponentially amplified by a DNA polymerase with strand-displacement activity. This mechanism enables RCA to generate long, single-stranded DNA products, which can be advantageous for various downstream applications, such as DNA sequencing or in situ hybridization techniques.
▪ Sensitivity: In certain scenarios, RCA has demonstrated higher sensitivity compared to PCR. Due to its isothermal nature, RCA can produce a larger number of amplification products, resulting in increased sensitivity for detecting low-abundance targets. This sensitivity advantage has proven particularly useful in applications such as detecting rare genetic mutations, single-cell analysis, or amplifying targets with low copy numbers.
▪ Simplified Workflow: RCA offers a simplified workflow compared to PCR. RCA reactions can be performed under isothermal conditions, eliminating the need for sophisticated thermal cycling equipment. This simplification can reduce overall costs and technical complexity associated with amplification procedures, making RCA an appealing option for resource-limited settings or POC applications.
▪ Product Length: As mentioned earlier, RCA can generate long, single-stranded DNA products. This feature proves advantageous in applications where longer DNA fragments are desired, such as generating templates for DNA sequencing or studying DNA–protein interactions. In contrast, PCR typically produces shorter amplicons due to limitations inherent in the polymerase enzyme and primer design considerations.
▪ Enzyme Selection: RCA can be conducted using various DNA polymerases, including both strand-displacing and nick-translating enzymes. This flexibility allows researchers to choose an appropriate enzyme based on their specific requirements, such as amplification efficiency, fidelity, or compatibility with specific detection methods. In contrast, PCR primarily relies on thermostable DNA polymerases, which may have limitations in certain applications, such as amplifying challenging templates or incorporating modified nucleotides.

While RCA offers several advantages over PCR in certain applications, it is important to note that PCR still maintains its strengths and remains the method of choice for many diagnostic and research purposes. PCR benefits from a well-established protocol, extensive commercial availability of reagents, and a vast body of literature supporting its applications. The selection between RCA and PCR should be based on the specific requirements of the experiment, the desired output, and the available resources. Additionally, the most recent studies focused on this accurate detection (specificity) using RCA method are summarized in Table 6.

## 5. Attraction of Antimicrobial Peptide (AMP) for *E. coli* O157:H7 Detection

The novel antimicrobial compounds have great potential for combating antibiotic resistance in bacteria. As part of the innate immune system, there is a growing demand for these compounds. To address this need, researchers have employed several alternative techniques, including the use of metals, metal oxides, polymer materials, and AMPs [60]. Among these techniques, AMPs show impressive potential for direct detection of both pathogenic and nonpathogenic bacteria. They are also considered strong candidates for whole bacteria detection. AMPs can be found in various natural niches and act as the organism’s first line of defense against bacterial attacks, forming an important part of the unique immune system that protects hosts from pathogens [61]. Most AMPs consist of 6 to 50 positively charged amino acid residues and a large number of hydrophobic residues [62]. Due to their excellent stability and low cost, AMPs have been extensively explored as alternative recognition elements in biosensors for bacterial detection. Since the affinity of AMPs is important in detection, the elements that influence the affinity of AMPs will be discussed before addressing various AMP-based approaches. Microfluidics have also been combined with AMP-based approaches to identify foodborne infections, making it a promising new tool. Finally, the future opportunities and challenges in constructing reliable and sensitive AMP-based platforms will be highlighted.

Although the mechanisms of action of AMPs have not been fully elucidated, there is a widely accepted model that explains their mode of action. According to this hypothesis, cationic AMPs bind to bacterial surfaces through electrostatic and hydrophobic interactions, leading to various modifications in membrane structures and disruption of the integrity of the bacterial cytoplasmic membrane, ultimately killing microorganisms. Based on their secondary structures, AMPs’ bioactivity toward microbial cells can be categorized into many groups [63]. Many AMPs adopt amphipathic conformations that allow them to target the negatively charged head groups of lipids on the bacterial membrane, utilizing spatial hydrophobic clustering from cationic amino acids. Plant and animal membranes, on the other hand, isolate negative charges from the interior leaflet and contain cholesterols that inhibit AMP activation [64]. AMPs, as antibiotics, have exhibited remarkable resistance to acquired resistance by targeting the fundamental structure of the bacterial cell membrane, making them difficult for proteases to identify. Linear cationic peptides such as magainin, with their small molecular size and inherent stability, are particularly attractive for microbial sensing applications [65]. As a precursor to bactericidal activity, the positively charged AMP magainin I (GIGKFLHSAGKFGKAFVGEIMKS) exhibits selective binding to the pathogenic bacteria *E. coli* O157:H7 [66,67]. Magainin I also demonstrates broad-spectrum efficacy against other Gram-negative bacteria responsible for a majority of human pathogenic infections.

In AMP-based biosensors, AMPs are often immobilized on substrate surfaces. The immobilized AMPs are believed to interact with the outer membrane through electrostatic contacts, triggering various interactions with the cytoplasmic membrane such as amphipathic conformations and insertion into the membrane, leading to disturbances in the lipid bilayer. Immobilizing peptides may restrict their mobility and ability to interact with cellular membranes compared to their soluble state. However, due to limited research on soluble and immobilized AMPs, it is challenging to determine the similarities and differences in their activities. The strong affinity of AMPs for bacterial surfaces has garnered more attention in biosensing applications than their antibacterial activity because AMP surfaces serve as recognition elements. The AMPs adsorbed on the surface of biosensors are believed to facilitate bacterial attachment through electrostatic and hydrophobic interactions. However, few studies have focused on the interactions of immobilized AMPs with the bacterial surface in biosensors. Despite the unknown recognition processes, AMPs have gained significant attention in biosensing due to their strong affinity for bacteria. The AMP database currently contains over 3000 natural AMPs, with their sequences and structures extensively studied by the research community.

One major advantage of AMPs as recognition elements is their ability to engage with multiple pathogens, thanks to their selective binding nature to target cells. By replacing existing antibody-based probes with more durable and stable antimicrobial peptides in biological sensors, the shelf life of current diagnostic systems can be extended. Due to these advantages, AMPs have been utilized to construct various bacterial biosensors. Numerous researchers have worked on developing microfluidic-based AMPs with specific identification elements for rapid and accurate detection of *E. coli* O157:H7 [68,69].

**Figure 4:**
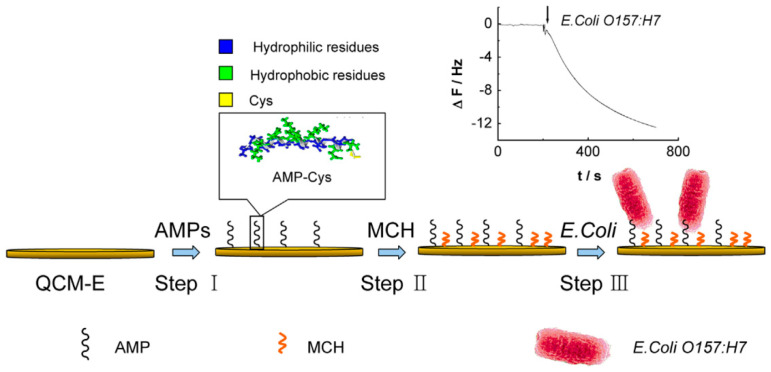
The principle of the binding reaction in the case of AMP-immobilized microfluidic biosensor for *E. coli* O157:H7 detection. (Step I) The AMPs with C-terminal are immobilized on a gold QCM electrode surface. (Step II) The surface is blocked with mercaptohexanol (MCH) to inhibit unnecessary sites for enhancement of the signal−to−noise ratio. (Step III) Implementation and detection of *E. coli* occur. Reprinted with permission from [70].

Various recognition elements, including magainin I, leucocyte A, clavA, colicin V, and cecropin P1, have been employed for bacterial detection. Among them, magainin I has shown the most promising binding results for reliable detection. Until recently, the majority of AMP research had focused on magainin II, but magainin I has proven to be a suitable bacterial recognition factor. Manoor et al. [69] demonstrated that immobilizing AMP magainin I on a gold microelectrode array via a C-terminal cysteine residue enabled the capture and measurement of the binding effect on pathogenic *E. coli*. They developed a robust and portable biosensor for the detection of *E. coli* O157:H7 using impedance spectroscopy for evaluation. The biosensor achieved a detection time of 30 min and a LOD of 1000 CFU/mL in water samples, with a detection range of 10^1^ to 10^5^. While this detection time is favorable, the LOD, which is a key factor for POC devices, needs improvement. To further improve the LOD and detection time, Dong and Zhao [70] fabricated the microfluidic *E. coli* biosensor with an excellent detection time and a lower LOD of 400 CFU/mL, which is 2.5 times better than the previous study [69]. They demonstrated that an AMP specific to *E. coli* O157:H7, tagged with a cysteine residue at the C-terminal for immobilization on a gold quartz crystal microbalance (QCM) electrode surface, could be used to capture and measure the binding effect. The complete detection mechanism is shown in Figure 4. While these approaches have achieved low bacterial cell detection limits, many of them require extensive sample preparation and, in some cases, multiple reagent and assay steps before detection. Therefore, developing an integrated detection system with high mobility, robustness, sensitivity, and selectivity for harmful organisms remains challenging. Further exploration is needed to improve the detection time and LOD for the identification/detection of *E. coli* O157:H7.

**Figure 5:**
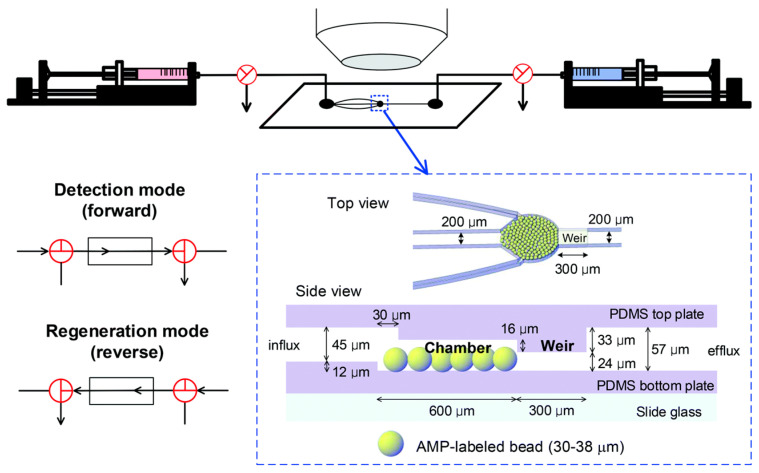
Illustration of a microfluidic biosensor based on AMP-embedded glass microbeads for *E. coli* detection, featuring both detection and regeneration modes. The weir structure of the microfluidic system plays a crucial role in facilitating the proper binding of *E. coli* on the glass microbeads. Reprinted with permission from [68].

**Figure 6:**
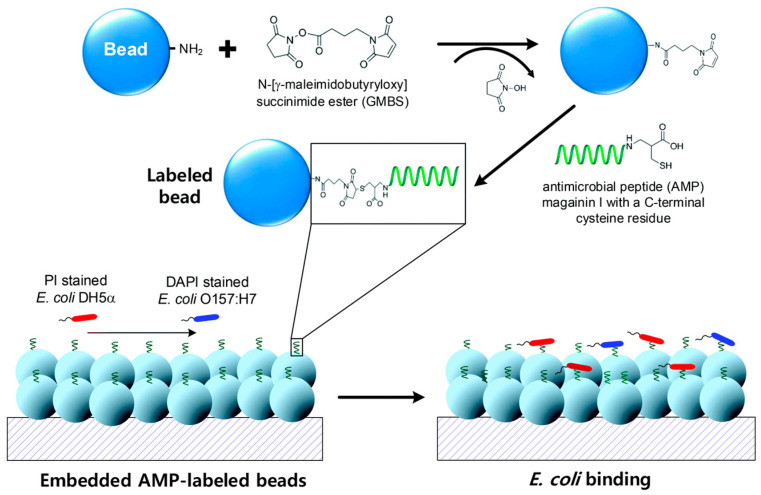
Schematics of AMP magainin I, with a C-terminal cysteine residue labeled onto the surface of glass microbeads, demonstrating the specific binding of both PI-stained nonpathogenic bacteria (*E. coli* DH5α) and DAPI-stained pathogenic bacteria (*E. coli* O157:H7) to the AMP-labeled beads. Reprinted with permission from [68].

To further enhance the LOD and decrease detection time, our research group [68] investigated the detection of pathogenic *E. coli* O157:H7 using a microfluid-based biosensing device embedded with AMP-labeled microbeads. Through effective immobilization of AMP magainin I on the surface of glass microbeads, the LOD as well as the detection time were significantly improved. We achieved an outstanding LOD of 10 CFU/mL with a detection time of less than 20 min. In addition, we successfully detected the entire bacteria, which is crucial for sensing pathogenic bacteria. Furthermore, we explored this microfluidic platform for effective regeneration capabilities. The strategic fabrication of our system is illustrated in Figure 5, highlighting the importance of the weir structure in the microfluidic channel. Figure 6 provides an explanation of the binding mechanism between the entire *E. coli* O157:H7 and the AMP-immobilized glass microbeads.

Most recent studies for the *E. coli* detection based on AMPs structure are summarized in Table 7 with the LOD and detection time. Further explorations are still needed for the identification/detection of *E. coli* O157:H7 to improve the detection time and LOD.

The accurate detection of pathogenic bacteria is a critical issue for POC devices to ensure better therapy for the corresponding disease and early-stage control. Currently, significant efforts are being directed towards achieving accurate and highly specific detection. To address this, Bai et al. [71] developed a sensor for the precise detection of *E. coli* O157:H7 using copper phosphate nanocomposites embedded with AMP magainin I and cecropin P1. The highly specific detection of *E. coli* O157:H7 was evaluated against multiple bacterial samples, with a bacterial density of 10^5^ CFU/mL (*n* = 3). In addition, Qi et al. proposed a novel microfluidic biosensor for the colorimetric detection of foodborne pathogens [80], and Zhang et al. introduced an integrated colorimetric and photothermal lateral flow immunoassay based on bimetallic Ag–Au urchin-like hollow structures for the sensitive detection of *E. coli* O157:H7 [80,81]. The most recent studies focused on this accurate detection are summarized in Table 8.

## 6. Conclusions and Future Challenges

This review provides an overview of recent advancements in the design and development of POC microfluidic-based biosensors for the detection of pathogenic bacteria, especially *E. coli* O157:H7. These biosensors offer a valuable means of delivering clinically relevant information in a simple, rapid, and cost-effective manner, catering to the demands of POC testing. The LOD and detection time are crucial parameters for detecting these pathogenic bacteria at an early stage of infection. While many of the proposed systems may not match the accuracy of traditional analytical methods, they can still provide sufficient information for routine testing and screening of food and water samples. This review summarizes the detection of *E. coli* O157:H7 using popular POC methods, such as SPR, RCA, and electrochemical methods. In addition, the impressive role of AMPs in terms of LOD and detection time is evaluated. Developing a simple, fast, and cost-effective method for *E. coli* detection not only ensures the safety of water and food supplies for consumers but also prevents costly recalls.

AMPs have emerged as a promising alternative to antibodies, and in particular, AMP-based microfluidic platforms for bacteria detection have garnered significant attention due to their simplicity, sensitivity, and high selectivity. In summary, while POC testing still has room for improvement, microfluidic biosensors hold great promise as practical tools for real-world applications. However, there are limitations in terms of LOD, detection time, and specific detection.

Improving the LOD, detection time, and accuracy of microfluidic chips is crucial. Additionally, it is equally important to detect the entire bacteria rather than solely focusing on its DNA or antibodies in order to save time and costs. AMPs present a viable option as they enable rapid and accurate detection of the entire bacteria. However, AMP-based detection faces challenges in terms of repeatability. Therefore, it is necessary to improve repeatability, along with the LOD, detection time, and selectivity, in order to develop efficient and useful biosensors for detecting pathogenic bacteria. Finally, in Table 9, a comparison is provided for widely applied methods used as POC devices for the detection of pathogenic *E. coli* O157:H7. Both the advantages and disadvantages of each method are summarized in this table.

## Figures and Tables

**Table 1:** Summary of recent studies for detection of *E. coli* O157:H7 using SPR methods.

Authors, Year	Materials	LOD/Detection Range (CFU/mL)	Detection Time (min)	Antibody (Ab)	Sample Type	Sensors Response
Li et al., 2021 [27]	SERS based organic and inorganic hybrid Au/Fe^3+^ nanoclusters	~2/1–10^6^	~30	monoclonal rabbit Ab	Spiked food	-
Yaghubi et al., 2020 [28]	LSPR based Au nanoparticle conjugated (non-covalent bond) with specific chicken anti-*E. coli* O157:H7 antibody	~10/10–10^5^	~120	chicken Ab	Chicken sample	530–543 nm
Kaushik et al., 2019 [25]	Functionalized 2D nanomaterial (MoS_2_ nanosheets)	~94/10–8 × 10^3^	~15	*E. coli* monoclonal Ab	Water and orange juice	637–644 nm
Zheng et al., 2019 [29]	Capture antibodies modified with magnetic nanoparticles (MNPs) and aggregation of Au nanoparticles; calorimetric biosensor	~50/50–5 × 10^8^	~30	Ab	Chicken sample	-
Lee et al., 2018 [30]	LSPR based magnetic nanoparticle coated with Au shell	~10/0–10^6^	~60	anti–*E. coli* O157:H7	Fresh lettuce	535–547 nm
Zhou et al., 2018 [31]	Silver nanoparticles-reduced graphene oxide (AgNPs-rGO)	~5 × 10^2^/10^3^–5 × 10^7^	~40	AMP with magainin 1-C	Water and juice	645–680 nm
Song et al., 2017 [32]	Immobilization of antibodies on Au NRs and Au NRs@SiO_2_	10/0.5–5 × 10^7^	~70 (+10 shaking)	murine anti-*E. coli* O157:H7 monoclonal Ab	PBS	718–775 nm
Lísalová et al., 2016 [33]	Ultra-low fouling and functionalizable poly(carboxybetaine acrylamide) brushes	17 & 57/1–10^8^	~80	Ab	Hamburger and Cucumber	0.0977 ± 0.03 nm
Tokel et al., 2015 [24]	Disposable microfluidic chips with Au coated surfaces functionalized with antibody	10^5^/10^5^–3.2 × 10^7^	~20	Ab	PBS and peritoneal dialysis fluid	Angle shift of 0.01
Tawil et al., 2012 [34]	T4 bacteriophage	10^3^/10^2^–10^5^	~20	T4 bacteriophage	PBS	0–35 pixels
Wang et al., 2011 [35]	MNPs modified with capture antibodies and aggregation of Au nanoparticles	3 × 10^4^/3 × 10^4^–3 × 10^8^	~10	rabbit anti-goat IgG polyclonal Ab	-	-

**Table 2:** Summary of recent studies for accurate detection of *E. coli* O157:H7 via SPR methods.

Authors and Year	Accurate Detection Results
Li et al., 2021 [27]	*E. coli* O157:H7 > *E. coli* > *S. typhimurium* > *S. aureus* > *V. parahemolyticus*
Zheng et al., 2019 [29]	*E. coli* O157:H7 > *Salmonella typhimurium* > *Listeria monocytogenes* > *non-target bacteria*
Zhou et al., 2018 [31]	*E. coli* O157:H7 > *non-pathogenic E. coli K12* > *Staphylococcus aureus* > *hemolytic streptococcus*
Tokel et al., 2015 [24]	*E. coli* O157:H7 > *Staphylococcus aureus* and *E. coli*> *Staphylococcus aureus*
Wang et al., 2011 [35]	*E. coli* O157:H7 > *non-pathogenic E. coli DH5α*

**Table 3:** Summary of recent studies for detection of *E. coli* O157:H7 via electrochemical methods.

Authors, Year	Materials	Assay Approach	Methodology	LOD/Detection Range (CFU/mL)	Detection Time (min)	Sample Type
Qaanei et al., 2021 [39]	Nanocomposite of reduced graphene oxide, Au nanoparticles and polyvinyl alcohol	Aptasensor	Differential pulse voltammetry (DPV)	17 (cucumber), 57 (hamburger)/9.2–9.2 × 10^8^	~100	Tap water, milk, meat
Zheng et al., 2021 [40]	Gold nanoparticles	Photoelectro chemical aptasensor	Electrochemical impedance spectroscopy (EIS)	200/0–4 × 10^7^	~40	Water
Ropero-Vega et al., 2021 [41]	Au nanoparticles-modified screen-printed electrodes	Bioinspired peptide in TIR protein as recognition molecule	Cyclic voltammetry (CV), EIS, Square wave voltammetry (SWV)	2/0–10^3^	~30	PBS
Li et al., 2020 [42]	Platinum nanoparticles	Aptasensor	Hybridized chemical reaction amplification	400/10^2^–10^7^	~15	Real milk
Park et al., 2021 [43]	PDMS-based finger-actuated microfluidic modules with Au electrodes	Geno-sensor	SWV	100/0–10^6^	~40	DI water, milk
Dhull et al., 2019 [44]	NiO/ITO electrode based immunosensor	Immunosensor	Amperometric biosensor, CV	1/10–10^7^	~60	Real milk
Chen et al., 2016 [45]	Magnetic nanoparticles and Au nanoparticles	Immunosensor	EIS	1.6 × 10^2^/10^2^–10^5^	~60	Spiked lettuce
Wang et al., 2013 [46]	Au nanoparticles modified graphene paper	Impedimetric immunosensor	EIS	1.5 × 10^2^/1.5 × 10^2^–1.5 × 10^7^	~30	Ground beef, cucumber
Altintas et al., 2018 [47]	Au nanoparticles amplified immunoassays	Impedimetric immunosensor	CV	50/10–3.97 × 10^7^	~35	Water
Yao et al., 2018 [38]	Magnetic nanoparticles, Au nanoparticles	Impedimetric immunosensor	EIS	12/12–1.2 × 10^5^	~15	Water
Yang et al., 2016 [48]	Au nano-films	Magneto-impedance sensor	Giant-magneto impedance effect	50/0–10^3^	~20	Water
Bai et al., 2020 [49]	Au nanoparticles	Aptasensor	CV, EIS, DPV	10/10–10^6^	~60	Spiked milk
Li et al., 2020 [50]	Hairpin primes and signal probes	Geno-sensor	CV, EIS, DPV	7/0–10^4^	~40	Apple juice, milk
Li et al., 2017 [51]	Carbon nanotube	Geno-sensor	CV, EIS	1/0–10^4^	~45	Apple juice, milk

**Table 4:** Summary of recent studies for accurate detection of *E. coli* O157:H7 via electrochemical methods.

Authors and Year	Accurate Detection Results
Qaanei et al., 2021 [39]	*E. coli* O157:H7 > *non-pathogenic E. coli K12* > *E. coli* > *Pseudomonas aeruginosa* > *Staphylococcus aureus* > *Salmonella typhimurium*
Zheng et al., 2021 [40]	*E. coli* O157:H7 > *Salmonella* > *Staphylococcus aureus* > *E. coli*
Ropero-Vega et al., 2021 [41]	*E. coli* O157:H7 > *P. aeruginosa* > *PBS* > *Staphylococcus aureus*
Li et al., 2020 [42]	*E. coli* O157:H7 > *E. coli K12* > *Staphylococcus aureus> Buffer*
Park et al., 2021 [43]	*E. coli* O157:H7 > *B. cereus* > *S. enteritidis*
Dhull et al., 2019 [44]	*E. coli* O157:H7 > *Staphylococcus aureus* > *non-pathogenic strain of* *E. coli*
Chen et al., 2016 [45]	*E. coli* O157:H7 > *L. monocytogenes* > *Mixture*
Wang et al., 2013 [46]	*E. coli* O157:H7 > *E. coli DH 5α* > *S. aureus* > *L. monocytogenes*
Altintas et al., 2018 [47]	*E. coli* O157:H7 > *Salmonella* > *Shigella* > *S. aureus*
Yao et al., 2018 [38]	*E. coli* O157:H7 > *Salmonella typhimurium* > *Listeria monocytogenes*
Bai et al., 2020 [49]	*E. coli* O157:H7 > *S. typhimurium* > *S. aureus* > *L. monocytogenes* > *P. aeruginosa*
Li et al., 2020 [50]	*E. coli* O157:H7 > *Vibrio cholera O1* > *Salmonella* spp. > *S. aureus* > *Listeria innocua*

**Table 5:** Summary of recent studies for detection of *E. coli* O157:H7 based on RCA.

Authors and Year	Materials	Assay Approach	LOD/Detection Range (CFU/mL)	Detection Time (min)	Sample Type
Li et al., 2021 [55]	PDMS surface, PAMAM dendrimers, aptamer, padlock probe, primers	Aptasensor	10^3^–10^4^/10^2^–10^5^	~60	Orange juice, milk, PBS, iced tea, bottled water
Jiang et al., 2020 [53]	PAMAM dendrimers, signaling RCA, primers/probes	Aptasensor	80/10^2^–10^5^	~90	Orange juice, PBS, milk
Jiang et al., 2017 [56]	PDMS surface, PAMAM dendrimers, primers/probes	Aptasensor	10^2^/10^2^–10^5^	~60	Orange juice, PBS, milk
Sun et al., 2020 [54]	UiO66 consisting of cubic framework of cationic nodes (formed in-situ via hydrolysis of ZrCl_4_) and 1,4-benzenedicarboxylate linkers	CRISPR (clustered regularly interspaced short palindromic repeats) based biosensor	4 × 10^2^/1.3 × 10^2^–6.5 × 10^4^	~120	Spring water, skim milk, orange juice
Luo et al., 2020 [57]	Hairpin probes	Aptasensor	75/2 × 10^2^–2 × 10^5^	~90	Defatted milk
Zhang et al., 2021 [58]	Aptamer, padlock probe, T4 DNA ligase, phi29 DNA polymerase, primers	Aptasensor based on DNA hydrogel	4 × 10^3^/4 × 10^3^–4 × 10^5^	~30	Spiked milk
Guo et al., 2016 [59]	Encapsulated silver nanocluster assembled by RCA	Electrochemical sensor	31/37–3.7 × 10^6^	~80	Milk

**Table 6:** Summary of recent studies for accurate detection of *E. coli* O157:H7 via RCA methods.

Authors and Year	Accurate Detection Results
Li et al., 2021 [55]	*E. coli* O157:H7 > *non-target E. coli ATCC25922*
Jiang et al., 2020 [53]	*E. coli* O157:H7 > *E. coli ER2420* > *E. coli K12* > *Listeria innocua*
Jiang et al., 2017 [56]	*E. coli* O157:H7 > *E. coli ER2420* > *E. coli K12* > *Listeria innocua*
Sun et al., 2020 [54]	*E. coli* O157:H7 > *S. typhimurium* > *L. monocytogenes* > *V. parahemolyticus* > *S. Aureus* > *S. flexneri*
Luo et al., 2020 [57]	*E. coli* O157:H7 > *S. aureus* > *S. Typhimurium* > *L. monocytogenes* > *S. flexneri* > *E. coli ATCC25922* > *E. coli* *CMCC44102> E. coli ATCC35218*
Zhang et al., 2021 [58]	*E. coli* O157:H7 > *E. coli O6* > *S. typhimurium* > *S. aureus* > *L. monocytogenes*
Guo et al., 2016 [59]	*E. coli* O157:H7 > *Salmonella* > *Bacillus Subtilis* > *Listeria*

**Table 7:** Summary of recent studies for detection of *E. coli* O157:H7 based on AMP.

Authors and Year	Materials and Recognition Elements	Sensor Type	Technique	LOD/Detection Range (CFU/mL)	Detection Time (min)	Sample Type
Bai et al., 2020 [71]	Cu phosphate nanocomposites embedded by AMP magainin I and cecropin P1	Immunosensor	Glucose meter readout	10/10–10^7^	~90	Spiked milk
Ding et al., 2020 [72]	AMP magainin I (C-terminal) functionalized magnetic nanoparticles, consists of Fe_3_O_4_ core	Electrochemical sensor	Dynamic light scattering (DLS)	5/5–5 × 10^6^	-	DI water, tap water
Yang et al., 2019 [73]	MnO_2_ on photo electrode surface, AMP magainin I as recognition element	Electrochemical sensor	Photo electrochemical	3/10–5 × 10^6^	~30	Tap water, tomato juice
Qiao et al., 2017 [74]	AMP conjugated with horseradish peroxidase (AMP–HRP)	Immunosensor	UV–VIS spectroscopy	13/10^2^–10^5^	~45	Apple, ground beef
Qiao et al., 2017 [75]	AMP functionalized magnetic nanoparticles	Immunosensor	PCR and fluorescence spectroscopy	84 (apple juice), 233 (beef)/10–10^6^	~30	Spiked apple juice, beef
Jiang et al., 2015 [76]	Au interdigitated electrode arrays immobilized with AMP colicin V (ColV)	Impedimetric sensor	Impedance spectroscopy	100/10^2^–10^6^	~10	Water
Dong and Jhao, 2015 [70]	AMP tagging with C-terminal cysteine for immobilization on Au electrode	Electrochemical sensor	QCM, EIS	400/0–1.8 × 10^6^	~10	Water
Kulagina et al., 2006 [67]	Immobilization of AMP magainin I, cecropin P1, and parasin on microscope slide glass	Immunosensor	Fluorescent microscopy	5 × 10^4^/0–10^7^	~90	Chicken
Chang et al., 2015 [68]	Microchannel embedded with AMP magainin I labeled glass beads	Diamidino-2-phenylindole (DAPI) stained sensor	Fluorescence spectroscopy	10/10–10^6^	~20	PBS
Yoo et al., 2014 [77]	Microchannel embedded with AMP magainin I labeled glass beads	DAPI-stained sensor	Fluorescence spectroscopy	10^3^/10^3^–10^6^	~30	PBS
Li et al., 2014 [78]	Immobilization of AMP magainin I on Au surface via C-terminal cysteine	Impedimetric sensor	EIS	10^3^/10^3^–10^7^	~90	Water
Schwartz and Bercovici, 2014 [79]	Conjugation of AMP with horseradish peroxidase (AMP–HRP)	Electrophoretic sensor	Fluorescent microscopy	100–10^4^/1–10^8^	~60	Water
Mannor et al., 2010 [69]	Immobilization of AMP magainin I on Au microelectrodes via C-terminal cysteine	Impedance-based sensor	Impedance spectroscopy	10^3^/10–10^5^	~30	Water

**Table 8:** Summary of recent studies for accurate detection of *E. coli* O157:H7 via AMP.

Authors and Year	Accurate Detection Results
Bai et al., 2020 [71]	*E. coli* O157:H7 > Non-pathogenic *E. coli* > *Staphylococcus aureus* > *Listeria monosytogenes* > invertase nanocomposites and Fe_3_O_4_ nanocomposites
Ding et al., 2020 [72]	*E. coli* O157:H7 > *Staphylococcus aureus* > *E. coli*
Yang et al., 2019 [73]	*E. coli* O157:H7 > *Salmonella* > *Staphylococcus aureus* > *S. epidermidis* > *Listeria monosytogenes* > *P. aeruginosa* and *E. coli* DH5α
Qiao et al., 2017 [75]	*E. coli* O157:H7 > *S. Typhimurium* > *E. coli* DH5α > *E. coli* BL21 and *L. monosytogenes*
Qiao et al., 2017 [74]	*E. coli* O157:H7 > *S. Typhimurium* > *E. coli* DH5α > *E. coli* BL21 > *Listeria monosytogenes* and *V. parahemolyticus*
Chang et al., 2015 [68]	*E. coli* O157:H7 > *E. coli* DH5α
Li et al., 2014 [78]	*E. coli* O157:H7 > *E. coli* K12 > *S. epidermidis* and *B. subtilis*
Mannor et al., 2010 [69]	*E. coli* O157:H7 > *S. Typhimurium* > non-pathogenic *E. coli* > *Listeria*

**Table 9:** Comparisons with different methods used as POC detection for *E. coli* O157:H7.

Sensing Methods	Advantages	Disadvantages
SPR-based sensor	Label free detection Real-time monitoring Reduces assay development time Low amount of sample volume Continuous measurement	Higher non-specific binding Expense of sensor chips Expensive instrumentation Low adoptability Poor LOD for *E. coli* O157:H7
Electrochemical-based sensor	Small size Low cost Easy to handle High sensitivity Rapid detection Real-time monitoring Nontoxic materials	Cannot be recycled Short shelf life Limited temperature range Unstable voltage Unstable current
RCA-based sensor	Whole bacteria detection Good sensitivity (e.g., LOD ~ 30 CFU/mL) Better detection time High specificity	Expensive method Short shelf life Difficult to handle Large amount of sample volume Regeneration of chip is difficult
Effect of AMP magainin I	Cost effective Rapid detection Accurate detection High sensitivity (e.g., LOD ~10 CFU/mL) Whole bacteria detection More durable and stable results Ability to bind a variety of pathogens Long shelf-life	Ability of natural AMPs Toxicity for oral application

## Data Availability

Not applicable.
